# Surface Generated Acoustic Wave Biosensors for the Detection of Pathogens: A Review

**DOI:** 10.3390/s90705740

**Published:** 2009-07-20

**Authors:** María-Isabel Rocha-Gaso, Carmen March-Iborra, Ángel Montoya-Baides, Antonio Arnau-Vives

**Affiliations:** 1 Grupo de Fenómenos Ondulatorios, Departamento de Ingeniería Electrónica, Universidad Politécnica de Valencia, Spain; 2 Instituto Interuniversitario de Investigación en Bioingeniería y Tecnología Orientada al Ser Humano, Universidad Politécnica de Valencia, Spain

**Keywords:** biosensors, Surface Acoustic Wave (SAW), Love Wave, Acoustic Plate Modes (APM), pathogen agents

## Abstract

This review presents a deep insight into the Surface Generated Acoustic Wave (SGAW) technology for biosensing applications, based on more than 40 years of technological and scientific developments. In the last 20 years, SGAWs have been attracting the attention of the biochemical scientific community, due to the fact that some of these devices - Shear Horizontal Surface Acoustic Wave (SH-SAW), Surface Transverse Wave (STW), Love Wave (LW), Flexural Plate Wave (FPW), Shear Horizontal Acoustic Plate Mode (SH-APM) and Layered Guided Acoustic Plate Mode (LG-APM) - have demonstrated a high sensitivity in the detection of biorelevant molecules in liquid media. In addition, complementary efforts to improve the sensing films have been done during these years. All these developments have been made with the aim of achieving, in a future, a highly sensitive, low cost, small size, multi-channel, portable, reliable and commercially established SGAW biosensor. A setup with these features could significantly contribute to future developments in the health, food and environmental industries. The second purpose of this work is to describe the state-of-the-art of SGAW biosensors for the detection of pathogens, being this topic an issue of extremely importance for the human health. Finally, the review discuses the commercial availability, trends and future challenges of the SGAW biosensors for such applications.

## 1. Introduction

Pathogenic agents such as bacteria, fungi and viruses are found widely distributed in the environment, food, marine and estuarine waters, soil and the intestinal tracts of humans and animals. Many of these organisms have an essential function in Nature, but certain potentially harmful micro-organisms can have profound negative effects on both animals and humans, costing the food industry (and indirectly, the consumers) many millions of dollars each year [1]. It is estimated that infectious diseases cause about 40% of the approximately 50 million total annual deaths world-wide [2]; waterborne pathogens cause 10–20 million of these deaths and, additionally, more than 200 million people each year, suffer non-fatal infections [3]. These facts have increased the need for more rapid, sensitive, selective, portable, power-efficient and low cost methods of detecting these pathogens. Biosensors offer a great potential for achieving this goal.

A biosensor can be defined as an analytical device in which a biologically active component (receptor), such as an enzyme, an antibody, etc., is immobilized onto the surface of an electronic, optic or optoelectronic transducer, allowing the detection of target analytes in complex mixtures [4]. Thus, advances in biosensing can be achieved by efforts in two main fields: the transduction mechanism and the biological reception mechanism (sensitive film). This fact makes biosensing highly interdisciplinary.

Biosensors may be divided into four basic groups – optical, mass, electrochemical and thermal – depending on the method of signal transduction. Acoustic Wave biosensors are mass sensors which operate with mechanical acoustic waves as their transduction mechanism. Acoustic Wave devices can be classified into three groups depending on the acoustic wave guiding process [5]: Bulk Acoustic Wave (BAW) devices, Surface Acoustic Wave (SAW) devices and Acoustic Plate Mode (APM) devices. In BAW devices the acoustic wave propagates unguided through the volume of the substrate, in SAW devices the acoustic wave propagates, guided or unguided, along a single surface of the substrate and in APM devices the waves are guided by reflection from multiple surfaces.

Traditionally, the most commonly used acoustic wave biosensors were based on a Thickness Share Mode (TSM) device [6], better known as Quartz Crystal Microbalance (QCM), which are classified as BAW devices. This was primarily due to the fact that QCM has been studied in detail for over 50 years. Therefore, it has become a mature, commercially available, robust and affordable technology [7,8]. Nevertheless, some acoustic wave devices based on SAW and APM, which operate efficiently in contact with liquid media (SH-SAW, LW, STW, SH-APM, LG-APM), have been reported as more sensitive than the typical QCM biosensors.

SAW devices were firstly used as filters and resonators in electronics and communications. Lately, they have called the attention of the scientific community for sensing applications. SAW devices are able to operate at higher frequencies than QCMs [9] and the acoustic energy of these devices is confined at their surface [10]. Higher frequencies lead, in principle, to more sensitive instruments because the acoustic wave penetration depth into the adjacent media is reduced [11]; this makes them very sensitive towards any changes occurring on the substrate surface, such as mass loading, variations of viscosity and conductivity [9]. Even, the FPW devices, which are operated at lower frequencies, have also been reported as very sensitive for biosensing. This particular case will be described in Section 4.3.

The first application of SAW devices as sensors was in 1979 for gas detection [12–14]. Later, in the 80s, early attempts to transfer the simple method of SAW gas sensing to a biosensor were less successful [9,15,16]; this was because these devices did not operate efficiently in contact with liquids. In some SAWs, particle displacements (Rayleigh waves) are normal to the surface of the device, which radiates compresional waves into the liquid and causes severe attenuation and high insertion losses. To avoid the high damping caused by the aqueous environment, the acoustic waves must be either shear horizontally polarized or have a phase speed less than the speed of sound in the liquid. The first successful approaches using SAW devices in contact with liquids were not achieved until 1987 [17,18]; these SAW devices operated with shear horizontal polarized waves. Another approach for facing this problem was the use of APM devices (SH-APM and FPW), which have been reported to work efficiently in liquid media. Thus, in the last 20 years, SAW and APM devices have called the attention of the biochemical scientific community for biosensing applications. Nowadays, SAW devices can be used to detect proteins, sugars, DNA, viruses, bacteria and cells [19]. APMs have also been reported for DNA [20], biomolecules [21,22], immunoreactions in complex biological media [23] and bacteria [24] detection.

The SAW and APM devices can be grouped as Surface Generated Acoustic Wave (SGAW) devices [25], because both develop acoustic waves generated and detected in the surface of the piezoelectric substrate by means of Interdigital Transducers (IDTs). Thus, these devices have many operation principles in common. This review provides a deep insight in SGAWs technology focused on biosensing applications. It describes the SGAWs operation principles for biosensors: measurement techniques, associated electronics and configuration set ups. It also offers a description of the different SGAW devices which can operate efficiently in liquid media and their state-of-the-art as biosensors for the detection of pathogen agents. Finally, the review discuses the commercial availability, trends and future challenges of the SGAW biosensor technology for such applications.

## 2. SGAW Basic Operation

**Figure 1.**
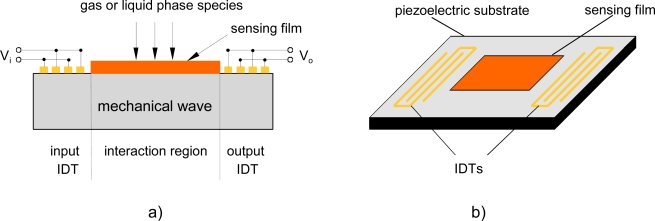
a) Structure of a SGAW sensor. b) IDT configuration for SGAW.

SGAW devices have been utilized as chemical sensors in both gaseous and liquid media. The input port of a SGAW sensor, comprised of metal electrodes (IDTs) deposited or photodesigned on an optically polished surface of a piezoelectric crystal, launches a mechanical acoustic wave into the piezoelectric material due to the inverse piezoelectric phenomenon and the acoustic wave propagates through the substrate (Figure 1). Biochemical interactions at the sensor surface cause changes in the properties of the acoustic wave (wave propagation velocity, amplitude or resonant frequency). These changes can be detected with network analyzers, vector voltmeters or more simple electronics, such as oscillators. The dimensions and physical properties of the piezoelectric substrate determine the optimal resonant frequency for the transmission of the acoustic wave [26].

The dielectric constant ε is an important parameter for the selection of the piezoelectric substrate material. If the sensor is to be operated in an aqueous solution of the analyte, ε should be close to that of water (*ε_r_* ≈ 80) in order to minimized a capacitive shortcut of the electrical field at the IDTs [27]. The most commonly used piezoelectric materials for these sensors are quartz (SiO_2_), lithium tantalate (LiTaO_3_), lithium niobate (LiNaO_3_), zinc oxide (ZnO) and aluminium nitride (AlN). Nevertheless, langasite has also been used in some investigations [28].

SGAW devices are highly sensitive to mass changes at the substrate surface; however, they are also sensitive to physical variables such as: polymer modulus, electric conductivity, and liquid density and viscosity [29], temperature, mechanical stress [11]. This review is focused on biosensing applications, which are mainly related to changes due to mass variations (mass sensitivity).

### 2.1. Interdigital Transducers (IDTs)

**Figure 2.**
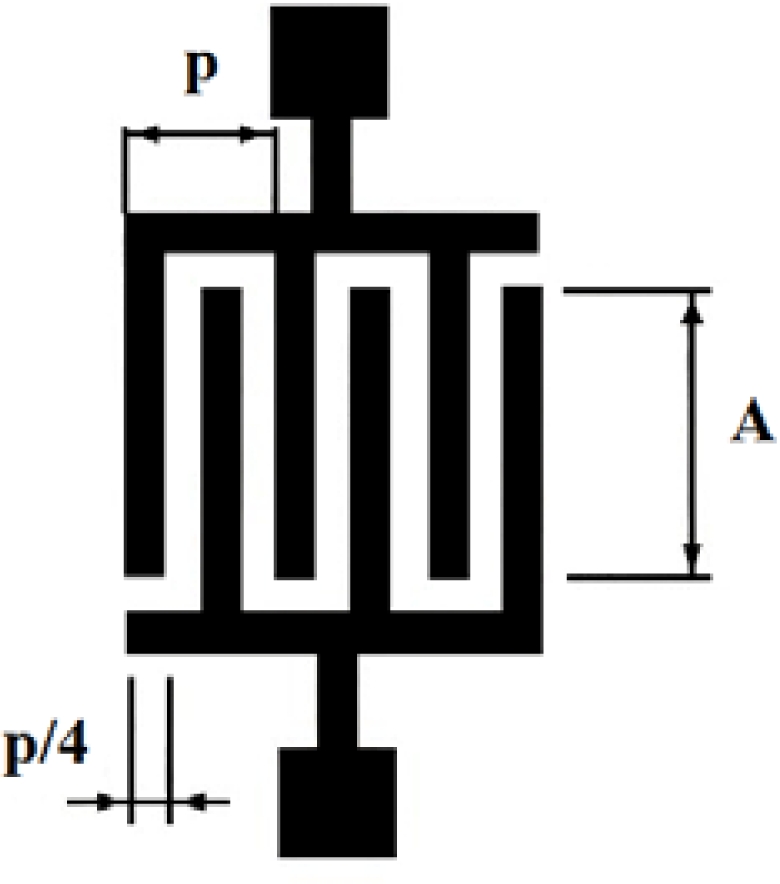
Interdigital Transducer (IDT) with period *p*, electrode width equal to space between electrodes and aperture A.

IDTs were firstly reported in 1965 by White and Voltmer [30] for generating SAWs in a piezoelectric substrate. An IDT, in its most simple version, is formed by two identical combs-like metal electrodes whose fingers are located in a periodic alternating pattern (see Figure 2). One comb is connected to the shield (ground) and the other to the center conductor of a coaxial cable where a radio frequency (rf) signal is provided.

Each transducer finger may be considered to be a discrete source for the generation of surface waves in a piezoelectric medium since the stress varies with position near each transducer finger. The spatially periodic electric field produces a corresponding periodic mechanical strain pattern by the piezoelectric effect. This gives rise to the SGAW which radiate in both directions away from the transducers orthogonally to the electrodes [29].

For an applied sinusoidal voltage, the transducer operates more efficiently when the SGAW wavelength, λ, matches the transducer periodicity, *p*, defined as the center-to-center distance between two consecutive fingers of one comb of the IDT. This occurs when the transducer is excited at the *synchronous frequency (f_o_*) in which all vibrations interfere constructively; where *f_o_* = *v_o_* */ p*, with *v_o_* the SGAW propagation velocity.

**Figure 3.**
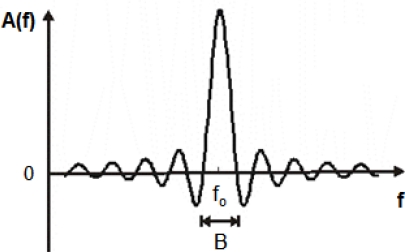
Frequency response of an IDT (positive frequencies).

The bandwidth *B* (Figure 3) of an IDT will be narrower when increasing the number of finger pairs *N*. However, there is a limitation in the maximum *N* recommended, due to the fact that, in practice, when *N* exceeds 100, the losses associated with mass loading and the scattering from the electrodes increase. This neutralizes any additional advantage associated with the increase of the number of the finger pairs; for example, the IDT impedance is affected by this parameter [31].

Due to symmetry of the IDT in the direction of propagation, the SGAW energy is emitted in equal amounts in opposite directions. This results in an inherent minimum of a 3 dB conversion loss for the transducer at *f_o_* [32].

### 2.2. SGAW Device Electronic Configurations

#### 2.2.1. Delay line - two IDTs configuration

**Figure 4.**
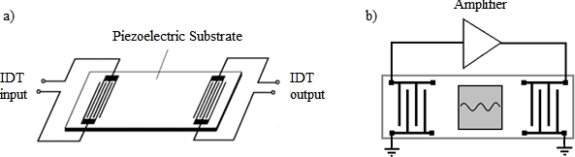
a) Two IDTs SGAW configuration. b) Two-port SGAW delay line oscillator. The SGAW device provides a feedback path for the amplifier. For a stable oscillation the signal must return to its starting point having equal amplitude and being shifted in phase by an integral multiple of 2π radians [31].

This is the most common configuration of SGAW biosensors. It consists of two IDTs (input and output). The input IDT launches a mechanical acoustic wave into the piezoelectric material, the acoustic wave propagates through the substrate and the output IDT receives the mechanical wave after a delay of some finite time and transforms it into an electrical signal due to the direct piezoelectric effect (Figure 4a). A sensitive layer, coated with a biological reagent such as an antibody, is placed on the surface of the device between the IDTs. When the sensitive layer is exposed to the particular antigen, a quantifiable change occurs in the amplitude and velocity of the propagating wave, which correlates to mass changes at the substrate surface [33]. Acoustic absorbers at both ends of the delay line reduce the spurious effects due to waves being reflected at the ends [34].

Most of the SGAW-based biosensors systems reported in literature consist of oscillator delay lines (Figure 4b), for which easy resonance frequency detection is not possible in most cases, since the device do not feature a single defined resonance frequency [9]. Delay line devices also suffer from high insertion losses since the acoustic wave has to travel a long distance on the surface of the device. In addition, placing a sealed cell in between the two IDTs produces drift, extra noise and generates lack of stability. Furthermore, a surface delay line produces an additional unwanted output signal known as the triple-transit signal, due to surface waves traversing the device three times because of acoustic reflections [35].

#### 2.2.2. Resonators

**Figure 5.**
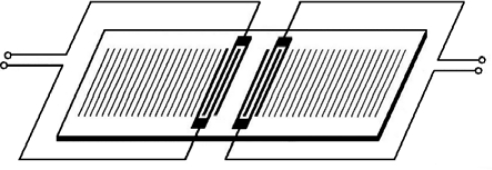
Scheme of a two-port SGAW resonator.

**Figure 6.**
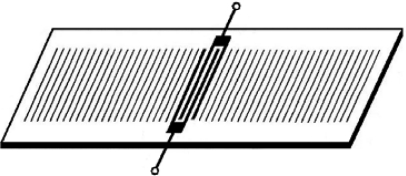
Scheme of a one-port SGAW resonator.

Two-port devices can be readily operated as resonators. These devices have four components, which are input and output IDTs and two sets of reflectors which form a resonant acoustic cavity (Figure 5). The second IDT must be located a few acoustic wavelengths from the launching transducer. It is also possible to operate with one-port SGAW resonators (Figure 6).

SGAW resonators are well established as one pole and narrow-band filters in a wide frequency range. Resonators offer the advantages of relatively high Q value, a low insertion loss, linear phase response and a higher frequency stability for a given device size, due to the multiple reflections which increase the group delay [36,37]. Moreover, SGAW resonators feature much lower insertion losses and are more suited for the detection of resonance frequency shifts via an oscillator circuit. Furthermore, these devices are compact and rugged and can significantly contribute to the reduction in the complexity of the system [37]. However, SGAW resonators suffer for considerable attenuation due to viscosity [38] and are very sensible to manufacture processes [9]. For this reason, SGAW resonators are not very common for research in biosensing applications; though, some research groups have worked with them [39–41].

#### 2.2.3. Dual-channel delay line

**Figure 7.**
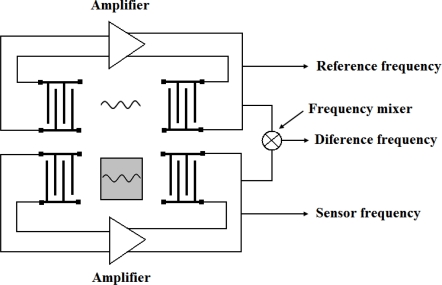
Dual-channel delay line configuration [46].

This configuration consists on having two identical delay line oscillators (that can be fabricated on the same substrate), one for sensing, in which the recognition agent is placed, and the other one as reference, where there is not such recognition agent. Common environmental interactions responses from both delay lines are removed by subtraction [42] (Figure 7). This setup is used to give a comparison of specific and non-specific responses as well as to eliminate the effects of viscosity and other disturbing influences such as temperature variations [34]. It also provides a signal whose frequency is easier to measure to high resolution, with simple frequency counters, than the frequency of the oscillation signal [43]; this reduces the complexity of the measuring electronic system. However, some authors state that a differential measurement technique cannot completely eliminate the temperature interference and the signals of the dual channel are also easily cross-talking to make the primary noise [44]. For dual-channel delay line oscillators where both delay lines reside on the same substrate, elastic waves can induce cross coupling and synchronization can occur [45]. Other researches stated that this setup does not significantly improve the measurement, at least not for the few minutes the sensor takes to respond [27], though they do not explain the reason.

### 2.3. SGAW Measurement Techniques

**Figure 8.**
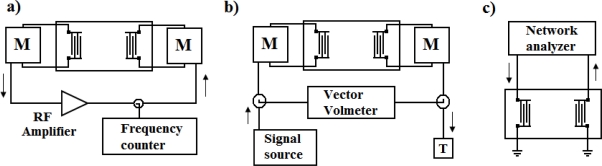
(a) Oscillator circuit provides a single-frequency signal. (b)Vector voltmeter provides phase and amplitude. (c) Network analyzers are connected to one and two-port devices. M: matching network [29].

The three basic electronic configurations for characterizing piezoelectric devices are shown in Figure 8.

#### 2.3.1. Oscillator

**Figure 9.**
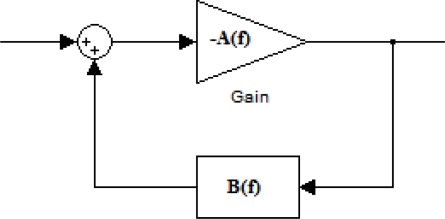
Feedback system for an oscillator.

The oscillator circuit places a two-port delay line SGAW piezoelectric device in the feedback loop of an rf amplifier (see *B(f)* in Figure 9). The condition for the oscillation is *A(f)*·*B(f)* = −1, from which |*A(f)*|·|*B(f)*| = 1 is found for the loop gain and for the loop phase arg *A(f)*·*B(f)* = −2π*n*, where *n* is an integer [32].

The oscillation frequencies of a SGAW oscillator delay line are given by *f_n_* = *n* · *v* / *L*, where *L* is the acoustic path length and *v* is the phase velocity. Relative changes in wave velocity lead to equivalent relative changes in oscillation frequency, Δ*f* / *f_o_* = Δ*v* / *v_o_*, where *f_o_* and *v_o_* are the unperturbed oscillation frequency and wave velocity, respectively, and Δ*f* and Δ*v* are the shifts in frequency and velocity respectively. Thus, the modification of the phase of the delay line due to mass changes can be measured as frequency shifts. Counting the oscillator frequency with a digital frequency counter provides a very precise indirect measurement of the acoustic wave velocities [29].

The oscillator configuration is the easiest electronic setup and the one that is most commonly used to determine the resonance frequency of a device. They can be designed to work with two-port and one-port devices. However, the drawbacks of oscillators are that they do not provide information about signal amplitude, they can be quenched if insertion losses exceed the amplifier gain during an experiment and if the amplifier operates in saturation, produces a distorted output signal containing many harmonics that may need to be filtered before counting the frequency [29].

#### 2.3.2. Vector voltmeter

This configuration consists of a signal generator, a two-port device and a vector voltmeter (Figure 8b). An rf voltage at a fixed frequency equal to the synchronous frequency of the device is provided by the signal generator to the input IDT. The changes in signal amplitude and phase shifts between the input and output IDTs are monitored by the vector voltmeter. Changes in phase indicate changes in wave velocity, while changes in amplitude indicate the attenuation of the wave.

The advantage of this electronic setup is that it provides velocity and amplitude information about the signal and avoids the disadvantages of a limited amplifier gain. On the other hand, phase measurements with commercially available vector voltmeters are 10 to 100 times less sensitive to velocity changes than frequency measurements by the oscillator setup. It is also possible to use a vector voltmeter in a phase-locked loop. In this case the phase is maintained constant by adjusting the frequency and the changes in frequency can be monitored [29].

#### 2.3.3. Network analyzer

Network analyzer is the instrument of choice to measure frequency responses of either one- or two-port devices, due to the fact that this setup allows a complete characterization of the devices under all conditions, including those for which the oscillator method fails. For two-port devices (see Figure 8c) the network analyzer records the transmitted signal to obtain the impedance characteristics of the device. Therefore, it works in the same way that a signal generator/vector voltmeter configuration being possible to measure amplitude and phase information of the signal as a function of the input frequency. Frequency scans can also be made during experiments to determine the device response as a function of time [29].

### 2.4. Acoustic Wave Particle Displacements

**Figure 10.**
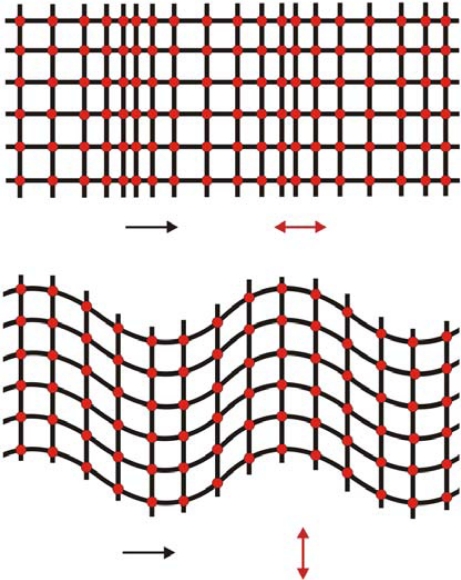
Top view of particle displacements of plane acoustic waves propagating in a solid. (Top) longitudinal or compressional wave. (Bottom) shear or transverse wave. Black arrows indicate the wave propagation direction and red arrows indicate the particle displacement directions.

Acoustic waves can be classified either for the particle displacement relative to the propagation direction of the wave (longitudinal or transverse) or for the particle displacement relative to the device surface (vertical or horizontal) [19]. The particle displacement of *longitudinal waves* (or *compressional waves*) is parallel to the wave propagation direction, while the particle displacement of *transverse waves* (or *shear waves*) is perpendicular to the wave propagation direction (Figure 10). The particles displacements of a *vertical wave* are normal to the surface of the devices, while the particles displacements of a *horizontal wave* are parallel to the surface. For a further understanding of particle displacement of an acoustic wave in a solid see references [31,47].

### 2.5. Crystal’s Cuts and Axis Rotation

**Figure 11.**
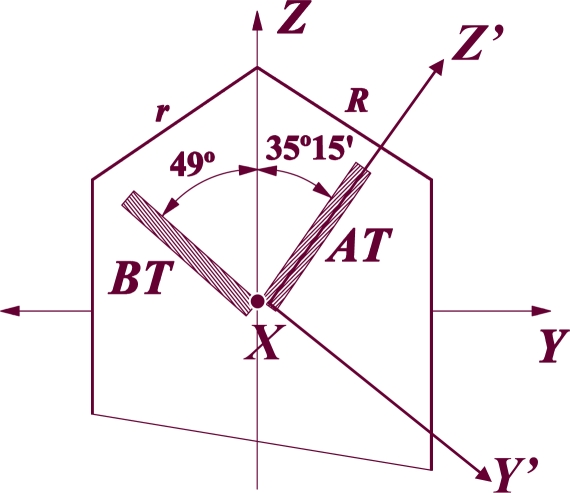
Y’-cuts of a quartz crystal (AT cut is 35°15′ rotated about the X- axis and BT cut is 49° rotated about the X- axis).

A Y’-cut is defined as a cut which has been rotated *θ* degrees about the original crystallographic X-axis of a crystal. In the same way, the X’-cuts are the ones rotated *θ* degrees about the Y-axis. As example, the most common Y’-cuts of quartz crystal are represented in Figure 11. The rotation about the Z-axis does not receive a particular name. Depending on the degrees of rotation about the three axes the elastic, dielectric and piezoelectric constants of a crystal change (see reference [48] for further understanding and rotation equations).

In SGAW literature cuts that are used to designate the direction of the normal to the major faces are often found. An X-cut has the normal to its major faces parallel to the X-axis of the crystal. In the same way, the Y- and Z cuts have their faces perpendicular to the Y- and Z-axes, respectively. In this type of cuts the elastic, dielectric and piezoelectric constants remain the same than the original crystal, because it has not been rotated (*θ* = 0°).

Other notation commonly used in literature to specify the crystal cut and features of a piezoelectric substrate is: degrees of rotation – cut type – wave propagation direction – substrate material. For example: “36° YX LiTaO_3_” means a 36 degrees rotated (with respect to the crystallographic X-axis), Y’-cut, X propagating lithium tantalate substrate.

## 3. SGAW Devices for Biosensing

Limitations of BAW devices arise from the fact that the improvement of sensitivity of these devices depends on the thickness of the piezoelectric substrate. In these devices, it is necessary to decrease the crystal thickness in order to operate at higher frequencies, which in general increases the device sensitivity. This makes BAW devices more complex to work with at higher frequencies than SAW devices. APM devices have the same disadvantage than BAW devices; sensitivity of these devices increases while decreasing substrate thickness. However, APM devices can operate at higher frequencies than common BAW devices.

Through appropriate selection of the substrate material, the substrate cut and orientation of the IDTs relative to the substrate, plate thickness and wave guiding mechanism, a variety of acoustic wave devices can be designed. In this review we just focus on SAW and APM devices (SGAW devices) that can be used as biosensor (devices which operate efficiently in contact with liquids). The direction of particle displacement at the surface of the device determines whether an acoustic wave device can be operated in contact with liquids or not. Vertical waves radiate compresional waves into the liquid, which causes severe attenuation (except in the case of the FPW device which is going to be treated later on). SGAW devices are manufactured using conventional IC microfabrication techniques, even CMOS processes [49]. This means that active signal processing could also be incorporated into the sensors.

### 3.1. Shear-Horizontal Surface Acoustic Wave (SH-SAW)

**Figure 12.**
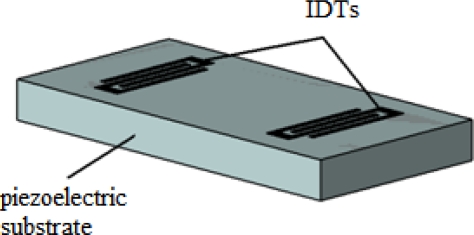
Scheme of a SH-SAW device.

In order to operate efficiently in applications which require fluid immersion, the Shear Horizontal Surface Acoustic Wave (SH-SAW) sensor was developed (Figure 12). This was the first sensor which used leaky waves, where the wave is only partially confined to the surface. The leaky-SAW mode is mainly shear horizontal but not purely shear horizontal and consequently suffers extra attenuation under fluid immersion [50]. Moreover, this wave extends several wavelengths into the device and therefore has a low sensitivity to changes at the device surface.

SH-SAW devices can be used for measurements in both liquid and gas media. The parameter that determines the resonance frequency of this device is the IDT spacing and its typical operating frequency is 30–500 MHz. The most commonly used substrate for this device is 36° YX LiTaO_3_, but ST- quartz [15,51], 41° YX LiNbO_3_ [52,53], 64° YX LiNbO_3_ [54–56], potassium niobate (KNbO_3_) [57] and Langasite (LGS) [28,58] have also been utilized.

For measurements in water an additional problem arises due to the dielectric constant of water (ε_r_ ≈ 80) which is significantly higher than that of quartz (*ε_r_* = 4.7). This leads to a dramatic decrease in the acoustoelectric coupling and to a significant electrical impedance mismatch which causes short-circuit of the IDTs through the water [59,60]. The later can be minimized by using substrate materials for the device with a *ε_r_* closer to that of water; for example, LiTaO_3_ (*ε_r_* = 47).

The major drawback of commercially available SH-SAW devices is the fact that the IDTs mostly consist of low-cost aluminum, so the lifetimes of such devices in aqueous media are limited to a few hours due to corrosion [9]. Therefore, additional protection layers are required like polyimide [61], Parylene C [62] or polystyrene [51].

### 3.2. Surface Transverse Wave (STW)

STW device operates with surface shear horizontal particle displacements, so it can be used for measuring in both gas and liquid media. The parameter that determines the resonance frequency of this device is the spacing of IDTs; its typical operating frequency is 30–300 MHz and the surface mass sensitivity reported in literature is 100–200 cm^2^/g [29,63]. The most commonly used substrate for this device is ST-cut quartz.

**Figure 13.**
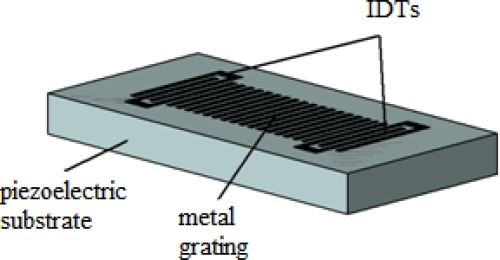
Scheme of a STW device.

A surface transverse wave is originated from a surface skimming bulk wave (SSBW) that travel very close to the surface but no exactly along it. A metal strip grating located in the surface of the devices between the input and output IDTs produces a slowing effect on the wave propagation velocity and traps the energy of the wave in the surface of the device enhancing its surface mass sensitivity (Figure 13). Thus, the STWs can be defined as grating-affected SSBWs.

**Figure 14.**
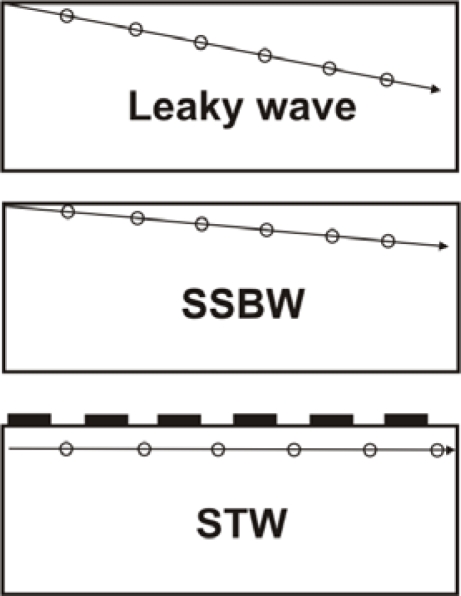
Wave propagation angles of Leaky, SSBW and STW waves.

The difference between leaky waves and SSBW waves is the wave propagation angle. Leaky waves have a larger propagation angle than SSBW waves. Figure 14 shows a scheme exemplifying the propagation angles of Leaky, SSBW and STW. As can be seen, Leaky waves have a higher propagation angle than SSBW and STW waves.

The SSBW was first described by Milson *et al*. [64] and Lewis [65] in 1977, but its advantageous qualities have turned out to be largely discredited by a substantial loss from radiation into the bulk of the substrate. It was not until 1987, when Bagwell and Bray [66] proposed a two-port resonator with an unloaded high quality factor (Q) of 5,600 in which the SSBW power was trapped to the surface via the effect of a metal strip grating.

Grating waveguides have certain advantages over plates. The grating can be matched to the transducer in order to prevent acoustic reflections and provides a stronger guiding [67]. They can also provide much higher sensitivity for the same thickness of material [68]. However, despite the numerous studies and results on STW resonant structures, little has been clearly said on how to achieve satisfactory quantitative understanding and prediction of device parameters [69]. In general, the inclusion of bulk losses appears to cause tremendous analytical difficulties. However, STW have proved to outperform conventional Rayleigh SAWs in a number of parameters. They are considerably faster, have a lower propagation loss and are more sensitive to outside impacts such as mass loading from absorbed gaseous substances. These qualities make the STWs suitable for a range of applications, including devices reaching the 3 GHz range and high sensitivity sensors [69].

### 3.3. Love Wave (LW)

**Figure 15.**
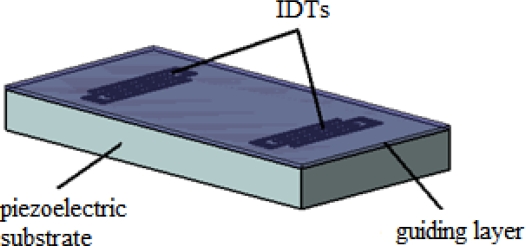
Scheme of a LW device.

Love wave devices (LWs) are comprised of a substrate that primarily excites a SSBW, which is subsequently confined by a thin guiding layer located on the top of the substrate and IDTs (Figure 15). Therefore, the IDTs remain isolated from liquids. The condition for the existence of Love wave modes is that the shear velocity of the overlay material is less than that of the substrate [43]. The waveguide layer confines the wave energy keeping it near the surface and slows down the wave propagation velocity. Thus, the LWs can be defined as layered-affected SSBWs. The sensitivity of a sensor is determined by the degree of wave confinement. If the wave is trapped tightly, it will be strongly perturbed by surface changes, yielding high sensitivity.

This device operates with a surface wave with shear horizontal particle displacements. Thus, it can operate efficiently in both gas and liquid media. The parameters that determine the resonance frequency are the spacing of IDTs and the thickness of the wave guiding layer. Typical frequencies in which this device operates are 80–300 MHz and the surface mass sensitivity reported for this device in literature is 150–500 cm^2^/g [29,63] .

Initially, the LWs were made in ST-cut quartz [70]; however, those devices lacked temperature stability, which is essential for field application. Thus, temperature-compensated systems based on different Y-rotated quartz and LiTaO_3_ plates were investigated [71]. Later, LiNbO_3_ substrates, like 64° YX LiNbO_3_ [72,73], were proposed for these devices, Waveguide materials such as polymers [74], silicon dioxide (SiO_2_) [71] and zinc oxide (ZnO) [75, 76] can be used for guiding layers [77].

### 3.4. Shear-Horizontal Acoustic Plate Mode (SH-APM)

This device was introduced in 1980s. It operates with a plate wave with shear horizontal particle displacements. Thus, this device can be used for measurements in contact with both gas and liquid media. The parameter that determines the resonance frequency of this device is the spacing of IDTs and the thickness of the substrate. The typical operation frequency of this device is between 25–200 MHz and the surface mass sensitivity reported in literature is 20–50 cm^2^/g [29,31,63]. The most commonly used substrates for this device are ST-cut quartz and ZX-LiNbO_3_. The SH-APMs have been used for measuring mass change in liquid media and also for detecting biologic molecules [21].

**Figure 16.**
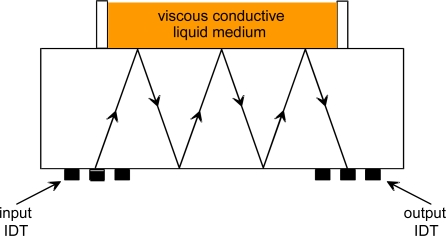
Structure of an SH-APM sensor.

The advantage of this device is that the IDTs can be located on the back side of the device and are thus away from the sensing side, what insolates the IDTs from the liquid (Figure 16). Thus, corrosion problems on electrodes resulting in deterioration of the sensor response are avoided. However, the main drawback of using SH-APMs is the fact that they are difficult to operate in a standard oscillator circuit. The reason for this is that several acoustic plate modes are usually excited simultaneously, but the frequency separation between these modes is often limited; which can produce a hopping mode in an oscillator circuit [9]. Additionally, mechanical and electrical loading of the surface can affect the APM sensor response; especially if a high-coupling piezoelectric material like LiNbO_3_ is used, the acoustoelectric interaction becomes important [22].

The use of APM delay lines has been hampered by the relative immaturity of the associated design techniques. The principle issue in the design of APM delay lines is to excite and detect electrically a single acoustic mode within the plate with low distortion from intermode interference or multiple waveguide reflections. The use of single-phase unidirectional transducers (SPUDT) enables the excitation and detection of a single acoustic mode, reducing the distortions that occur in conventional transducer designs [20].

Theoretically, the sensitivity of this device for an isotropic plate is given by *S_m_* = −*J* / *ρ*·*d*; where *J* = 1/2 for the mode *n* = 0 and *J* = 1 for higher plate modes (*n* > 0), *ρ* is the plate density and *d* is the plate thickness [29]. Decreasing the plate thickness increases the frequencies of higher plate modes of the SH-APM device and it also increases mass sensitivity. Thus, higher-order modes appeared to be more sensitive than lower-order modes, although they have more transmission losses [78].

### 3.5. Layer-Guided Acoustic Plate Mode (LG-APM)

McHale *et al*. [79,80] suggested, from theoretical considerations, that a guiding layer could be used on one substrate face of a SH-APM device to create a LG-APM in a similar way to Love waves and so obtain a sensitivity approaching that of a LW. They suggested that higher order Love modes can be regarded as continuations of the layer-guided SH-APM. The most commonly used substrates for this device are ST-cut quartz and 36° YX LiTaO_3_.

Evans *et al*. [81] demonstrated experimentally that lithium tantalate substrates could be used for a LG-APM, which exhibit an enhanced mass sensitivity compared to the traditional SH-APM. Thus, it is possible to retain the advantages of operating with liquids on the opposite face to the transducer as with the SH-APM device with a mass sensitivity enhancement.

This device operates with a plate wave with shear horizontal particle displacements. Thus, measurements in both, gas and liquid media are possible. The parameters that determine the resonance frequency are the spacing of IDTs and the substrate thickness of the guiding layer. Typical operating frequencies are 25–200 MHz and the surface mass sensitivity reported is between 20–40 cm^2^/g [29,63].

### 3.6. Flexural Plate Wave (FPW)

**Figure 17.**
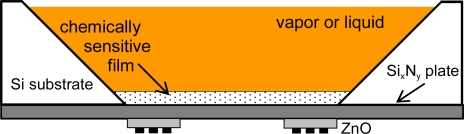
Scheme of a FPW device.

FPW devices are built with plates that are only a fraction of an acoustic wavelength thick (typically 2–3 mm). The confinement of acoustic energy in such a thin membranes results in a very high mass sensitivity. The plates are composite structures (Figure 17) consisting of a silicon nitride layer, an aluminum ground plane, and a sputtered zinc oxide piezoelectric layer, all of which are supported by a silicon substrate. This device operates with a plate wave with vertical particle displacements. Measurement in gas and liquid media are possible, due to the fact that FPW velocity is less than the compressional velocity of sound in liquids. Therefore, this device does not couple compressional waves in liquid and only minor energy dissipation occurs [31]. The parameters that determine the resonance frequency of this device are the spacing of IDTs and thickness of the substrate. Typical operation frequencies are 2–20 MHz and the surface mass sensitivity reported is 200–1,000 cm^2^/g [82]. When the device is operated in liquid, the mass sensitivity falls from 1,000 cm^2^/g to about 200 cm^2^/g, because of mass contributed by the liquid in the evanescent field region [67,83].

**Figure 18.**
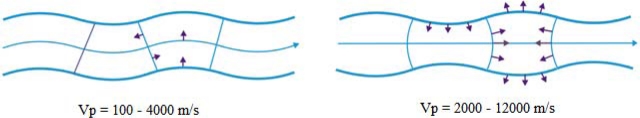
Pictorial representation of Lamb wave modes: (left) antysimmetric mode and (right) symmetric mode. Typical wave speeds, Vp, are shown below each sketch.

When the substrate thickness is less than the penetration depth, an interaction is produced between the guided modes in both substrate faces and the Lamb modes are then generated [31]. The particle displacements of a Lamb wave are similar to those of the Rayleigh wave, elliptical components both normal and parallel to the surface. In fact, a Lamb wave can be considered as being composed of two Rayleigh waves propagating on each side of a plate with a thickness of less than one wavelength. For plates thicker than a few wavelengths, two free Rayleigh waves propagate. Plate waves can propagate in symmetric or antisymmetric modes [84]. In Figure 18, the motions of groups of atoms of a Lamb wave are depicted in the cross-sectional view of the wave propagating to the right. Particle displacement directions are represented with red arrows. The wave velocity of the first antisymmetric mode (A_0_) is much lower than those of the other possible modes. A_0_ is the only mode that presents a phase velocity going to zero when the normalized thickness, *d*/*λ,* goes to zero; where *d* is the plate thickness and *λ* is the wavelength. This mode has flexible features; hence it is also known as Flexural Plate Wave (FPW).

The theoretical mass sensitivity of this device is given by S_m_= −1/2*ρd*, where *ρ* is the plate density and *d* is the plate thickness [29]. The mass sensitivity increases with decreasing plate thickness (decreasing frequency for a constant wavelength). Thus, the main advantage of flexural plate wave device (FPW) is its high sensitivity to added mass at a low operating frequency, which eases the associated electronics requirements. However, FPWs are fragile due to the reduced device thickness [85].

Other advantages of FPW sensors are their on-line, real-time performance, compatibility with aqueous samples, and variable surface chemistry. A particularly interesting feature of FPW is their potential for the sentinel activities in remote or inaccessible locations [86]. In addition, the FPW can be used to measure cell concentrations and growth rates in industrial fermentors, biofilms, and wastewater treatment facilities [87].

## 4. SGAW Biosensors for Pathogen Detection

Microbial and viral identification and quantification assays usually rely on conventional approaches of plating and culture methods, as well as on biochemical testing, microscopy, etc. Over the last 20 years, many new methods have been developed, including immunological methods, polymerase chain reaction (PCR) and biosensors [88]. Plating and culture methods often fail to provide the required specificity and sensitivity and in addition it takes a long time (up to 7 days). PCR, although very specific and suitable for screening purposes, still fails to produce accurate results when enumeration of viable cells is needed [89]. Immunological detection with antibodies is perhaps the most successful technology employed for the detection of cells, spores, viruses and toxins alike [90]. Polyclonal antibodies can be raised quickly and cheaply but they are often unspecific and available in limited amounts. In contrast, monoclonal antibodies (MAbs) have the advantage of ensuring reproducibility and permanent reagent supply [89]. The availability of MAbs, together with the emergence of recombinant antibody phage display technology, has made immunological detection of microbial contamination more sensitive, specific, reproducible and reliable [1]. These technologies, when incorporated in biosensors, significantly shorten the assay time and improve the analytical performance of pathogen detection. Biosensors can also be based on specific or non-specific protein interactions (with antibodies, antigens or enzymes), DNA hybridization or other biomaterials.

The immobilization of biomolecules on the solid substrate of the transducer surface is essential to ensure biosensor performance, because of its role in specificity, sensitivity, reproducibility and recycling ability. Among all of the immobilizing methods reported in the literature, covalent binding is the most promising technique since it allows retention of biological activity of biomolecules after immobilization. Covalent immobilization assures a reproducible, durable and stable attachment to the substrate against physico-chemical variations in the aqueous microenvironment. Self-assembled monolayer (SAM) technology provides the best results in covalent binding and allows the generation of monomolecular layers of biological molecules on a variety of substrates. Gold surfaces allow the use of functionalized thiols, whereas SiO_2_ surfaces enable the use of various silanes [4]. Both methods produce monolayers of active groups for the subsequent coupling of biomolecules onto the transducer surface. However, since no single immobilization method has proven to be optimum for all possible transducers [2], suitable immobilization methods have to be developed for every combination of biological reagent and sensor surface.

Piezoelectric devices represent a cost-effective alternative to other popular transducers for biosensors, such as advanced optical approaches [91]. Among piezoelectric biosensors, QCM-based applications have extensively been reviewed [10]. As regards SGAWs, some approaches to biosensors based on STW [4,68,92], SH-APM [20,22,23,93,94], and LG-APM [95] devices have been reported. However, none of these approaches address the detection of pathogens. Here, we present the state-of-the-art of piezoelectric SGAWs based on SH-SAW, LW and FPW biosensor transducers applied to pathogen detection.

### 4.1. SH-SAW

In 1987, Moriizumi *et al*. described the first biosensor application of SH-SAW transducers in liquid medium [18]. Further on, in 1993, Rapp. and coworkers suggested the use of commercially available SH-SAW filters for communication applications, with frequency ranges of 150–1,000 MHz and lithium tantalate (LiTaO_3_) as the substrate, to develop an immunosensor [96]. A similar approach was presented by these authors in 1995. They produced an immunosensor by covalent antibody immobilization, via the cyano-transfer technique, to a thin polyimide layer that preserved aluminium IDT’s of the transducer from corrosion when working in aqueous buffers [60].

More recently, Deobagkar and coworkers [51] developed a SH-SAW immunosensor for the detection of *E. coli* O157:H7 in water. In this immunosensor, polyclonal antibodies were covalently attached to polystyrene-coated active transducer surface. The authors reported a detection range of 0.4 –100 cells/μL giving, frequency shifts over 1.5– 5.8 kHz in an 87.7 MHz oscillator. This detection allowed the determination of up to 0.4 cells/μL of *E. coli* in water. Thus, the device was sensitive enough to detect this pathogen at concentrations which could cause human health hazards.

In a previous work, Berkenpas *et al*. [28] used a SH-SAW transducer fabricated on langasite (LGS) crystals to successfully detect macromolecular protein assemblies. This device demonstrated favorable temperature stability, biocompatibility, and low attenuation in liquid environments, suggesting its applicability to bacterial detection. Later on, the same authors applied and validated this previously reported LGS SH-SAW biosensor for the detection of *E. coli* O157:H7 [58]. They derivatized these LGS SH-SAW delay lines by attaching a biotinylated polyclonal rabbit antibody, directed against *E. coli*, to NeutrAvidin™ SAM functionalized gold surface.

In 2006, Länge *et al*. [97] presented a new approach to integrate a SH-SAW biosensor in a microfluidic polymer chip. The chip is easy to handle and its total volume is of only 0.9 μL. According to preliminary experiments with such microdevice, the authors stated that SGAW biosensing systems based on these chips promise fast response times and low sample consumption for bioanalytical sensing applications.

### 4.2. LW

The first approaches employing LW for biochemical sensing were reported in 1992 by Kovacs *et al*. [98] and by Gizeli *et al*. [99], who first demonstrated the use of such devices as mass sensing biosensors in liquids. However, it was not until 1997 that Harding *et al*. [100] used a LW acoustic device to detect real-time antigen-antibody interactions in liquid media. In 1999, Freudenberg and coworkers built a contactless LW device in order to protect electrodes from the conductive and chemically aggressive liquids used in biosensing [101].

In 2000, Howe and Harding [102] used a dual channel LW device as a biosensor to simultaneously detect *Legionella* and *E. coli*. In this approach a novel protocol for coating bacteria on the sensor surface prior to addition of the antibody was introduced. Quantitative results were obtained for both species down to 10^6^ cells/mL, within 3 h.

In 2003, Tamarin *et al*. [103] designed a LW immunosensor as a model for virus or bacteria detection in liquids (drinking or bathing water, food, etc.). They grafted a monoclonal antibody (AM13 MAb) against M13 bacteriophage on the device surface (SiO_2_) and sensed the M13 bacteriophage /AM13 immunoreaction. The authors suggested the potentialities of such acoustic biosensors for biological detection. The same year, Kalantar-Zadeh *et al*. [104] showed that mass sensitivity of LW devices with ZnO layer was larger than that of with SiO_2_ guiding layers. They monitored adsorption of rat immunoglobulin G, obtaining mass sensitivities as high as 950 cm^2^/g. The authors pointed out that such a device was a promising candidate for immunosensing applications.

Branch and coworkers reported in 2004 [105] a LW biosensor for the detection of pathogenic spores at or below inhalational infectious levels. A monoclonal antibody with a high degree of selectivity for anthrax spores was used to capture the non-pathogenic simulant *Bacillus thuringiensis* B8 spores in aqueous conditions. They suggested that acoustic LW biosensors will have widespread application for whole-cell pathogen detection.

Due to the fact that direct anti- *E. coli* antibodies grafting onto the sensor surface did not lead to a significant detection of whole bacteria, in 2007 Moll *et al*. [106] developed an innovative method for the detection of *E. coli* employing an LW device. It consisted of grafting goat anti-mouse antibodies (GAM) onto the sensor surface and introducing *E. coli* bacteria mixed with anti-*E. coli* MAb in a second step. The sensor response time was shorter when working at 37°C, providing results in less than 1 hour with a detection threshold of 10^6^ bacteria/mL. More recently, the same authors [107] described a multipurpose LW immunosensor for the detection of bacteria, virus and proteins. They successfully detected bacteriophages and proteins down to 4 ng/mm^2^ and *E. coli* bacteria up to 5.0 × 10^5^ cells in a 500 μL chamber, with good specificity and reproducibility. The authors stated that whole bacteria can be detected in less than one hour.

Taking into account that SGAW biosensors are a powerful tool for the study of biomolecular interactions, Andrä *et al*. [108] used a LW sensor to investigate the mode of action and the lipid specificity of human antimicrobial peptides. They analyzed the interaction of those peptides with model membranes. These membranes, when attached to the sensor surface, mimic the cytoplasmic and the outer bacterial membrane.

Finally, Bisoffi *et al*. [109] used a LW immunosensor to detect Coxsackie virus B4 and Sin Nombre virus (SNV), a member of the hantavirus family. They described a robust biosensor that combines the sensitivity of SGAW at a frequency of 325 MHz with the specificity provided by monoclonal and recombinant antibodies for the detection of viral agents. Rapid detection (within seconds) for increasing virus concentrations was reported. The biosensor was able to detect SNV at doses lower than the load of virus typically found in a human patient suffering from hantavirus cardiopulmonary syndrome.

### 4.3. FPW

White *et al*. and White and Wenzel first reported in 1987–1989 the use of FPW dispositives as sensors in gaseous [82] and liquid [83,110,111] environment. By the same time, Constello *et al*. [112] monitored the changes in frequency and viscosity of such devices over time, when proteins adsorbed onto the sensor surface.

However, it was not until 1998 that Pyun *et al*. [24] described an approach of a biosensor for *E. coli* employing an “acousto-gravimetric” FPW transducer. They covalently coupled antibodies, against *E. coli* K12 and *E. coli* J5 bacteria, to the aminosilanized monolayer modified platinum active surface, and measured changes in frequency in a flow system. A detection range of 3.0 × 10^5^ to 6.2 × 10^7^ cells/mL was obtained. To further increase the sensitivity, they used microespheres coupled with anti *E. coli* antibodies in a sandwich assay, amplifying the signal about five-fold.

In 1999, Cowan *et al*. [87] performed an on-line real-time measurement of changes in the concentration of *E. coli* W3110. The authors detected those changes as the cells settled onto the sensor under the influence of gravity, and reported that experimental data were in good agreement with a developed theoretical model for sensor’s response to cell settling. They postulated the use of FPW sensors as devices to measure cell concentrations and growth rates in industrial fermentors, biofilms and wastewater treatment facilities.

Recently, Kuznetsova and Coakley [113] stated, in a complete review article, that biosensors incorporating ultrasound standing wave systems (USW) can show enhanced sensitivity due to the effect of the direct radiation force (DRF) and induced acoustic streaming. DRF concentrates particles of the analytical sample and can also increase the capture rate by delivering suspended particles to immune-coated surfaces. Acoustic streaming provides rapid and homogeneous mixing in small analytical systems, otherwise difficult to achieve. Streaming induced in FPW immunosensors, and cavitation micro-streaming in the analytical cell, significantly accelerated the antigen-antibody reaction. The authors presented an example of such an ultrasound system to demonstrate *E. coli* K12 cell capture efficiency on the surface of immunomagnetic beads, stating that this phenomena can be implemented in FPW based sensors.

## 5. Commercial SGAW-Based Biosensors. Trends and Challenges

Most of the SGAW-based biosensor systems are just laboratory setups suitable for proof-of-principle evaluation and first experimental tests [9]. However, there are some close-to-commercial LW-based biosensors. The “S-Sens” (developed by the CAESAR research institute in Bonn, Germany) consists of a five channel LW device. Detection is performed by measuring changes in signal amplitude and phase. Experimental results obtained with this commercial biosensor have been reported by some groups [108,114–118]. Other commercially available system is the SAW-MDK1 (Senseor, Mougins, France), which consists of a microbalance development kit with two-channel delay lines based on LW devices, but no results derived from their applications have been reported yet. To our knowledge, there are no commercially available biosensors based on FPW devices. However, there are numerous patents of systems based on them [119–121].

Among SGAW devices, researchers seem to be more attracted by LW biosensors, since their features and the experimental results obtained point them as the most promising device for this purpose. It is clear that the commercial trend has been directed towards the use of LW for a more stable and efficient biosensor system, while the other SGAW devices setups have not get past the laboratory or the patent stages.

The big potential of the SGAW technology has not yet been very well recognized by the scientific community, according to the review presented in 2007 by Gronewold [10], where the number of publications referencing QCM, Surface Plasmon Resonance (SPR) and SGAW technologies were compared. This might be due to the technological hindrances found for applying this sensor for biosensing, or to the sensitivity of SGAWs to viscoelasticity. Reports about applications where mass alterations are separated from viscoelastic effects can enhance the acceptance of SGAW sensor technology.

Nowadays, the trend is the placement of multiple, small, versatile sensors into a network configured for a specific location. SGAW devices are moving into the lab-on-a chip arena. Nevertheless, SGAW technology still has some hurdles to clear [11]. SGAW biosensors packaging needs further development. The packages cost ten times more than the sensors they contain [68]. In addition, much research and effort are still required addressing the fluidic technology issue. Integration and automation with electronics and flow cells could reduce costs of the device and increase the throughput. For a better performance of SGAW sensors, the combination with other detection methods such as optical [25] or chromatographic [122] are being considered.

Mathematical modeling and simulations of these devices is also essential for the development of new sensors, especially with respect to the study of new materials and wave propagation [10]. Numerical calculations and Finite Element Method (FEM) analysis of SGAW devices have been reported in literature for further understanding of this topic [57,123–127].

## 6. Concluding Remarks

From the material discussed in this review, it seems that SGAW devices are promising for the measurement of interfacial biochemical phenomena. Although conventional methods for the detection and identification of microbial contaminants can be very sensitive, inexpensive, and able to provide both qualitative and quantitative information, they usually require several days to yield reliable results. Biosensors constitute a real alternative to traditional methods, since they can offer label-free, on-line analysis of antigen-antibody interactions and provide the option of several immunoassay formats, which allow increased sensitivity and specificity. When applied to the detection of bacteria in food and water, SGAW biosensors allow rapid, real-time, and multiple analyses, with the additional advantages of their cost effectiveness and ease of use.

Although SGAW-based sensor technology has been in the focus of academic and industrial research for more than 20 years, SGAW-based biosensors are neither well recognized nor well established in the market yet. The drawbacks associated with this kind of biosensors include relatively long incubation times of the bacterial sample on the biosensor surface, problems with crystal surface regeneration, high packaging cost, and difficulties to implement the related fluidic. SGAW technology for biosensing applications can be improved by devoting additional effort to flow cell design, system miniaturization, transducer sensitivity enhancement, and antibody design and production. Better oscillator circuitry, more temperature-stable substrates, and more stable device configurations should be pursued. The application of suitable effort and resources to these areas should result in fully automated, fast, less expensive and portable biosensing systems that could routinely be used in laboratory and field applications for the detection of micro-organisms with high sensitivity.

LW appears to be the most promising SGAW device for biosensing applications. In fact, LW-based biosensor systems are already commercially available. On the contrary, SH-APMs seem to have been left behind, since there are not updated references about biosensors based on these devices. Consequently, no detection of pathogenic agents using this approach has been reported. This might be due to its lower sensitivity and to its complexity of use. On the other hand, very few articles have been published about LG-APM biosensors and no detection of pathogens has been accomplished with this device either. SH-SAW is being clearly improved by STW and even more by LWs. STWs are more complex to manufacture than LWs due to the surface metal grating, whereas LWs can achieve higher sensitivities.

Compared to other methods, SGAW technology may not be adequate for clinical diagnosis in terms of sensitivity. Nevertheless, SGAWs could properly fulfill application requirements where a high number of measurements is needed and/or the detection limit is not so demanding.
